# The challenges of assessing patients' medication beliefs: a qualitative study

**DOI:** 10.1186/s12913-017-2020-y

**Published:** 2017-02-07

**Authors:** Rachael J. Thorneloe, Christopher E.M. Griffiths, Darren M. Ashcroft, Lis Cordingley

**Affiliations:** 1 Division of Pharmacy and Optometry, Manchester Academic Health Sciences Centre, University of Manchester, Manchester, M13 9PB UK; 2 Centre for Dermatology Research, Manchester Academic Health Sciences Centre, University of Manchester, Manchester, UK; 3 Salford Royal NHS Foundation Trust, Salford, UK; 4 Centre for Pharmacoepidemiology and Drug Safety, Division of Pharmacy and Optometry, Manchester Academic Health Sciences Centre, University of Manchester, Manchester, UK; 5 Division of Musculoskeletal and Dermatological Sciences, Manchester Academic Health Sciences Centre, University of Manchester, Manchester, UK

**Keywords:** Beliefs about Medicines Questionnaire, Adherence, Shared decision-making, Psoriasis, Necessity-Concerns Framework, Cognitive Interviewing

## Abstract

**Background:**

An estimated 50% of patients do not take their medication as prescribed, with medication adherence associated with adverse outcomes and higher costs of care. The Necessity-Concerns Framework identified individual’s beliefs about their medication as playing a key role in adherence, and UK Clinical Adherence Guidelines recommend eliciting and incorporating individual’s perceptions of their medication within the consultation. The Beliefs about Medicines Questionnaire (BMQ) is widely used to assess medication beliefs, however, given the condition-specific nature of some self-management regimens, it is unknown whether this tool is able to fully capture beliefs about more complex medication regimens.

**Methods:**

We examined the challenges of assessing medication beliefs using the BMQ in 20 people with a complex relapsing-remitting condition recruited from community sources. Data were collected from people with psoriasis; a patient group characterised by complex medication regimens, which include therapies that are applied topically, phototherapy/photochemotherapy, and therapies that are administered orally or via subcutaneous or intravenous injections. Semi-structured cognitive interviews were undertaken, with responses coded using established schedules and analysed using Content analysis.

**Results:**

Individual’s beliefs about their condition specific therapies were not accurately captured by the BMQ. Medication beliefs as expressed during ‘real-time’ completion of the BMQ were underestimated, or failed to be captured, by the corresponding scores given by participants.

There was mismatch between the terminology used in the scale and individuals perceptions of their condition and the complexity of its management and treatment outcomes. Currently the BMQ cannot represent beliefs about medicines underuse, even though some individuals with psoriasis viewed access to therapies as overly restrictive. Some the BMQ items were misinterpreted in part due to ambiguous item wording or due to misreading by participants.

**Conclusions:**

This is the first study to identify general and condition-specific difficulties experienced by individuals completing the BMQ in ‘real time’. The main implication of this research is the need to develop condition-specific versions of the BMQ in order that this important instrument can capture the full range of medication beliefs in individuals living with a complex relapsing-remitting condition. Access to condition-specific versions could significantly increase our understanding of beliefs which facilitate or reduce medication adherence.

## Background

The effectiveness of medical therapies reported in clinical trials are usually based on atypical levels of medication adherence. Whilst it is recognized that such outcomes may not be replicated in ‘real world’ clinical practice, patients need to be optimally adherent to their medication in order to achieve the best clinical outcomes. Adherence is defined as the extent to which the patient’s behaviour (medication usage) matches agreed recommendations from the clinician [1]. High levels of adherence is a crucial component of effective self-management for many people living with long-term conditions (LTCs) and viewed as one of the key mediators between medical practice and clinical outcomes. However adherence rates in those with LTCs are low, with people typically taking only half of their prescribed medication [2]. The World Health Organisation [3] views non-adherence as an important public health concern, with implications for treatment response [4], mortality [5], and additional healthcare costs, including increased number of hospital admissions and appointments, wasted resources, disease progression and need for more aggressive medications [1, 6].

The UK National Institute for Health and Care Excellence (NICE) Medicines Adherence Guidelines [7] recognise that non-adherence can be unintentional, referring to unforeseen barriers beyond the control of the patient, or intentional, in which the patient makes a deliberate decision not to follow the prescribed medication regimen. In order to support shared medication decision-making and adherence, they recommend that individual’s beliefs about their condition and medication should be appropriately elicited and incorporated by the clinician within the consultation. This approach has been shown to be effective in supporting medication adherence [8, 9]. According to the Necessity-Concerns Framework [10, 11], key beliefs that influence medication adherence are perceptions of personal need for medication for current and future health (necessity beliefs) and concerns about potential negative consequences. The Beliefs about Medicines Questionnaire (BMQ) [12] is the tool most commonly used to assess and quantify beliefs about prescribed medication and medicines in general. Items from the BMQ are also included in the NICE Guidelines as illustrative questions that could be asked to the patient in a consultation.

The BMQ was originally developed to assess commonly held beliefs representative of all illnesses [10, 11], however many medication regimens are condition-specific, with LTCs involving a range of different complex and sometimes unique medication challenges. It is unknown whether the BMQ can capture condition-specific beliefs. Indeed, the association between medication beliefs and adherence has not been demonstrated in all studies, and there are marked differences in effect sizes [13, 14]. Whilst it is clear that this variation may reflect differences in clinical populations, the contribution of other factors that influence adherence, and differences in the definition and assessment of adherence [13, 15], one important consideration is whether condition-specific beliefs are fully captured by existing BMQ items.

One example of a complex condition associated with considerable medication challenges is psoriasis, a common incurable inflammatory skin condition [16] affecting approximately 2-3% of the general population in the UK [17]. Although psoriasis can occur at any age, the majority of cases occur before the age of 30 years [18]. It is generally characterised by well-delineated thick, red and heavily scaled plaques and people have to manage considerable psychological and social morbidity [19, 20], complex medication regimens and adherence difficulties [21, 22]. Mild, localised disease may be treated with topical therapies (gel, ointment, lotion) by a general practitioner (GP; family doctor), whilst more severe disease may be managed in specialist dermatology (hospital) settings with phototherapy or photochemotherapy (light therapy) or traditional systemic (tablets) or biologic therapies (injections) that have immunosuppressant properties [23]. Typically individuals may move from one therapeutic approach to another. They may also be required to cope with concerns about unwanted medication adverse effects, uncertainty about the degree of disease control achieved by their medication, unpredictable relapsing and remitting symptoms, perceptions of incurability, inadequate understanding about the causes of psoriasis, widespread treatment dissatisfaction and a perceived lack of support from clinicians [24–29]. Unlike many other LTCs, there is evidence to suggest that a large proportion of people with psoriasis believe that their condition is not treated sufficiently aggressively [26].

In order to improve our understanding of the influences on medication adherence, the aim of this qualitative study was to examine whether items in the BMQ are able to fully capture the complexity of condition-specific medication beliefs, using psoriasis as an exemplar of a relapsing-remitting condition where complex medication challenges are present.

## Methods

### Participants

Using purposive sampling, people with a diagnosis of psoriasis who were 18 years or over were recruited from community venues (e.g. places of worship, libraries, community halls) and from a national established patient support association in England. No relationship with potential participants was established prior to study commencement. A participant information pack (PIS) was sent to 75 potential participants who responded to the study advertisement, with interested participants asked to contact the researcher to schedule an interview. The PIS provided information about the researcher and the reasons for the study. A separate study (different research questions and analytic methods) using the same participant sample has been published [22].

### Procedure

Cognitive interviewing (sometimes known as the ‘think-aloud’ approach) [30–32] is a technique whereby participants verbalise and report what they are thinking about as they solve a problem or complete a task, and has been used to examine the content validity of several questionnaires [33–36]. It can be used to identify how individuals experience completing a questionnaire and the degree to which items capture the constructs they purport to measure, and thus has an important role in the development and evaluation of questionnaires alongside other psychometric assessments.

Using this technique, participants were asked to ‘think-aloud’ and mark their chosen response option for each item of the BMQ. Participants were instructed to answer each item in response to their current prescribed therapy. For those using a combination regimen, participants were asked to complete the BMQ twice; they discussed the items in relation to their primary (phototherapy/photochemotherapy or systemic therapy) and secondary therapy (topical therapy) separately. Once they had completed the BMQ, participants were asked to suggest any other medication beliefs not included in the current version of the BMQ. The use of prompts was limited in order to allow participants to verbalise their own thought processes, however if it was necessary to further elucidate any problems or difficulties they identified when responding to questionnaire items, then a structured prompt was used (Table 1). Participants documented their demographic, medication and disease history before each interview. The recruitment procedures continued until data saturation was reached (no new themes emerging). Interviews were conducted during October 2011 and February 2012 at a location convenient for them; participants were reimbursed for any travel expenses but were not paid for participation. Only the participant and the interviewer were present during interviews; the mean duration was 90-min and were conducted, audio recorded verbatim and transcribed by one author (R.J.T.). The interviewer was a health psychology researcher with experience and training in qualitative methodology. The interpretative components were undertaken using secondary validation by a second author (L.C.). The multidisciplinary research team (health psychologist, dermatologist, and pharmacist), engaged in ongoing discussion to achieve consensus on data coding and to reduce interviewer bias and assumptions.

**Table 1:** Example structured prompts, adapted from Willis (2005) [32]

Suggested prompts	
•What does that term mean to you as it’s used in the question? •What were you thinking about when I asked that question? •How did you come up with that answer? •How easy or hard was it to determine your answer? •How did you decide on this answer? How did you decide on this category response? •How well does this category response apply to you? •Does the term used in this question sound OK to you, or would you use something different?	

### Materials

The BMQ [12] is an 18-item measure comprising of two subscales assessing beliefs about medication as whole, relating to the *nature* of medication (general harm) and beliefs about *how* they are used by doctors (general overuse), and two subscales assessing beliefs about medication prescribed for a specific condition, relating to perceptions about the *personal need* for medication (specific necessity) and *concerns* about potential negative effects from medication (specific concerns). The following are representative items in the BMQ-Specific subscale: ‘My life would be impossible without my psoriasis medication’ and ‘I sometimes worry about the long-term effects of my psoriasis medication’. Example items in the BMQ-General subscale are: ‘Doctors use too many medicines’ and ‘People who take medicines should stop their treatment for a while every now and again’. Satisfactory reliability and validity coefficients have been reported [12]. Each item is scored on a 5-point scale, ranging from ‘strongly disagree’ (=1) to ‘strongly agree’ (=5), with higher scores indicating stronger medication beliefs. The BMQ-Specific items were adapted so that the instructions referred to the participant’s prescribed psoriasis therapy. One additional item was included in the BMQ (‘Doctors use too few psoriasis therapies’) to assess whether participants believe access to psoriasis therapies is overly-restrictive.

### Analysis

Content analysis [37] was used to explore the challenges of assessing medication beliefs using the BMQ, by coding the experiences of responding to BMQ items. The a priori coding framework was based on schedules used in previous cognitive interview studies [34, 36] and was applied systematically to the data (Table 2). Data were analysed in three stages. First, data for each BMQ domain was analysed separately. Second, data were coded using the a priori coding framework. Third, data were analysed into themes that derived from the data, with these themes presenting the rationale for the initial data coding.

**Table 2:** Item response coding framework

Coding framework	
Code 1 Suitable question	No problems emerged
Code 2 Problematic item content	Participants identified problems with the terminology used or questioned the relevance of the question(s) to psoriasis or medication
Code 3 Misinterpretation	Participants answered a different question from one that was asked or their verbal responses to the question did not match the response option selected

## Results

A final sample of 20 people demonstrated interest and agreed to participant in the study, with 14 people recruited from a support website and 6 recruited from the community (Table 3).

**Table 3:** Demographic and disease characteristics of participants

Characteristic		*N*
Sex	Female	9
	Male	11
Ethnicity	White	18
	Indian	1
	Chinese	1
Employment	In paid work (full or part-time, including self-employed)	12
	In full-time education/training	3
	Unemployed	1
	Retired	3
	Retired and voluntary work	1
Education	1 or more ‘O’ level equivalents	1
	1 or more ‘A’ level equivalents	2
	Trade qualifications	3
	Professional qualifications	8
	Degree	6
Housing	Owner occupied/mortgaged	14
	Rented from local authority/housing association	2
	Rented from a private landlord	3
	Other (living with parents)	1
Psoriasis type	Chronic plaque	19
	Palmoplantar psoriasis	1
Co-morbid conditions	Psoriatic arthritis	3
Current therapies*	Topical	16
	Phototherapy/photochemotherapy	1
	Oral systemic	6
	Biologic	1
	Non-prescription (e.g. tanning booths, Chinese medicine)	3
Past therapies*	Topical	18
	Phototherapy/photochemotherapy	12
	Traditional systemic	7
		Median (range) in years
	Age	48.5 (21 – 71)
	Age when psoriasis started	14.5 (4 – 52)

Key: * = more than one characteristic can be recorded by each individual

### Specific necessity domain

Of the five specific necessity questions, one question (‘My psoriasis medications protect me from becoming worse’) did not generate problems relating to item content (Code 2) and two questions (‘My psoriasis medications protect me from becoming worse’ and ‘Without my psoriasis medications I would be very ill’) did not generate problems relating to misinterpretation (Code 3). The analysis produced four themes for the specific-necessity domain (Table 4).

**Table 4:** Thematic description for the specific necessity domain

Specific necessity domain		
Coding framework	Themes	Description
Code 2 Item content	Theme 1 Illness identity	Mismatch between the terminology used in the scale and participants perceptions of their psoriasis.
	Theme 2 Illness outcomes	Mismatch between the terminology used in the scale and participants perceptions of the complexity of psoriasis management.
Code 3 Misinterpretation	Theme 3 Medication side-effects	Participants mistakenly referred to potential medication adverse effects.
	Theme 4 Alternative medications	Participants mistakenly referred to obtaining alternative medications.

### Theme 1: Illness identity

Participants viewed psoriasis as being a separate and distinct entity from their perceptions of disease, with psoriasis described as a difficult and distressing ‘condition’, rather than a life-threatening illness. Participants objected to some of the terminology used in the specific necessity scale, in particular the terms ‘health’, ‘ill’ and ‘impossible’, because these terms did not match with their perceptions of psoriasis.

“I can understand why people would define psoriasis as an illness, but I suppose I tend to see it more as a *condition*.” (P4, specific necessity Q3 ‘Without my psoriasis medications I would be very ill’, item response ‘disagree’, score 2/5)

Some participants perceived their psoriasis medication as ‘necessary’, whilst others viewed medication as having little impact on physical comfort, psychological and social well-being. Due to the terminology used in the scale, these items could under-estimate medication necessity beliefs as expressed in the interviews, with some participants choosing to disagree with the specific-necessity statements. Thus, these questions could not differentiate responses given by individuals who believed that their psoriasis medication was necessary and those who did not.

“…It can be very sore, it can be very *itchy*, it can drive you mad…I would maybe be quite *depressed* about the whole thing, but I don’t think it would make me *ill*” (P14, specific necessity Q3 ‘Without my psoriasis medications I would be very ill’, item response ‘disagree’, score 2/5)

“My life wouldn’t be impossible, but it would be *more difficult* and I would feel socially a lot more conscious. And I’m sure without them my psoriasis would be a lot more worse than it is.” (P11, specific necessity Q2 ‘My life would be impossible without my psoriasis medications’, item response ‘disagree’, score 2/5)

### Theme 2: Illness outcomes

Participants thought that terms used in the BMQ items such as ‘health’, ‘life’ and ‘ill’ were too broad to capture the complexity of psoriasis management and questioned whether the terms were referring to the management of physical symptoms, psychological or social well-being.

“Well in fairness, do they mean *mental* health or physical health…?” (P7, specific necessity Q1 ‘My health, at present, depends on my psoriasis medications’, item response ‘disagree’, score 2/5)

Beliefs in whether psoriasis medications helped to manage physical symptoms or psychological/social well-being were not necessarily the same, however the BMQ does not differentiate between these different outcomes.

“…I think that the *social impact* that the physical symptoms would give me, as opposed to the physical symptoms themselves, would make me ill. It would probably make me very sad, very depressed.” (P11, specific necessity Q3 ‘Without my psoriasis medications I would be very ill’, item response ‘uncertain’, score 3/5)

### Theme 3: Medication side-effects

Due to the broad terminology used in the specific necessity scale (e.g. the term ‘health’), some participants misinterpreted the items about perceived need for psoriasis therapies for current and future health. Some mistakenly referred to the impact of potential medication adverse effects on future health, rather than discussing perceived need for psoriasis therapies for managing current and future psoriasis symptoms. This resulted in scores being an inaccurate representation of their medication necessity beliefs.

“I don’t know whether any of the *medications* will have impacted on any of my internal organs.” (P16, specific necessity Q4 ‘My health in the future will depend on my psoriasis medications’, item response ‘agree’, score 4/5)

### Theme 4: Alternative medications

Many people with psoriasis will use different types of psoriasis therapy over the course of their condition, due to inter-individual medication response and changes to the severity of the condition. Furthermore, medications can lead to adverse effects and efficacy can diminish over time, and so they can be administered as combination, rotation or sequential regimens [38]. As a result, some participants discussed the possibility of obtaining alternative psoriasis medications, rather than discussing their perceptions of current medication necessity. As a result, scores could again be an inaccurate representation of medication necessity beliefs.

“I’m uncertain about that, and the reason for that is because you never know what medications might be around the *corner* which might be able to help me.” (P11, specific necessity Q4 ‘My health in the future will depend on my psoriasis medications’, item response ‘uncertain’, score 3/5)

### Specific concerns domain

Of the five specific concern questions, one question (‘Having to take my psoriasis medications worries me’) did not generate problems relating to item content (Code 2) and one question (‘I sometimes worry about the long-term effects of my psoriasis medications’) did not generate problems relating to misinterpretation (Code 3). The analysis produced six themes for the specific concern domain (Table 5).

**Table 5:** Thematic description for the specific concerns domain

Specific concerns domain		
Coding framework	Themes	Description
Code 2 Item content	Theme 5 Terminology	Participants did not understand the term ‘mystery’.
	Theme 6 Medication outcomes	Mismatch between the terminology used in the scale and participants perceptions of the complexity of medication outcomes.
	Theme 7 Overly restrictive	Participants believed that access to psoriasis medications was overly restrictive, with this belief not assessed in the BMQ.
Code 3 Misinterpretation	Theme 8 Failure to reflect the degree of concerns	Participants stopped taking their medication because they were so worried about them. However they stated that they no longer had concerns because they had stopped taking their medication.
	Theme 9 Illness vs. medications	Participants misinterpreted the items and referred to their psoriasis.
	Theme 10 Uncertainty about prescribed medication	Participants chose the ‘uncertain’ response option to reflect their own uncertainty about their medication and how they worked, not uncertainty about their response.

### Theme 5: Terminology

Psoriasis therapies may conflict with patients’ understanding of the causes of their psoriasis; indeed, misunderstanding regarding the causation of psoriasis has been reported, including the belief that allergies [18] and uncontrollable factors (e.g. chance, back luck, poor medical care in the past, ageing) [39] are possible causal factors. In order to understand patient’s perceptions of their medication, assessing whether people understand why they are prescribed a particular therapy and how these medications work is necessary. Although item eight of the BMQ (‘My psoriasis medications are a mystery to me’) attempts to assess perceived understanding, participants did not understand the term ‘mystery’.

“I don’t understand that [mystery]…I don’t know quite how to take that” (P17, specific concerns Q8 ‘My psoriasis medications are a mystery to me’, item response ‘uncertain’, score 3/5)

### Theme 6: Medication outcomes

Participants expressed the view that some terms used in the specific concern statements were too broad; participants differentiated between short- and long-term medication side-effects, current and future medication disruptions to daily life, and between physical and psychological medication dependency. As it currently stands, the BMQ does not differentiate between these different outcomes.

“No I never worried about the long-term effects. It was the *short*-*term effects* which bothered me. The fact that it caused me irritation.” (P9, specific concerns Q7 ‘I sometimes worry about the long-term effects of my psoriasis medications’, item response ‘strongly disagree’, score 1/5)

“I get a bit *iffy* if I realise that I’ve not got another one of everything in the house. I once left my wash bag behind and it had everything in it… my shampoo, my scalp solution, my creams…two whole days without anything. So it is like being dependent, but more *mentally* dependent on it” (P15, specific concerns Q10 ‘I sometimes worry about becoming too dependent on my psoriasis medications’, item response ‘strongly agree’, score 5/5)

### Theme 7: Overly restrictive

A number of participants who were managed by their GP believed that access to psoriasis medications was overly restrictive. Existing BMQ items did not assess these beliefs and thus the inclusion of the condition-specific item (‘Doctors use too few psoriasis therapies’) was a useful addition to the BMQ.

“Oh yes, strongly agree [that doctors use too few treatments]. From my own experience from a number of G.P’s, I have found that they don’t know what treatments are available and they seem extraordinarily reluctant to use any, even if they are aware of them actually” [P4, over-restrictive belief Q19 ‘Doctors use too few psoriasis therapies’, item response ‘strongly agree’, score 5/5]

“Yes I do [agree that doctors use too few treatments]. I mean, they don’t even look into…even with all the things [therapies] coming out, they cannot be bothered to give you something else to try.” [P10, over-restrictive belief Q19 ‘Doctors use too few psoriasis therapies’, item response ‘agree’, score 4/5]

### Theme 8: Failure to reflect the degree of concerns

A number of participants had stopped using their medication because they were so worried about them. However participant’s medications concerns were not identified by the BMQ items, as they felt the statement no longer applied (that is, “*I no longer have concerns because I have stopped medication”).* This resulted in the underestimation of medication concerns. Those with very high levels of concern could give low scores, with these scores incorrectly indicating that they did not have concerns about the medication.

“You know, I could use it every day for 2 months, *but I don’t*. The principle reason, other than it being a pain, is the long-term effects and skin thinning…” (P6, specific concerns Q9 ‘My psoriasis medications disrupt my life’, item response ‘strongly disagree’, score 1/5)

### Theme 9: Illness vs. medications

When discussing medication concerns, some participants discussed their psoriasis rather than their psoriasis therapies, resulting in scores being an inaccurate representation of their concern beliefs.

“I suppose they do really because when it gets bad, I find it hard to not pick at it and *scratch it*. And there are bits of skin all over the place.” (P9, specific concerns Q9 ‘My psoriasis medications disrupt my life’, item response ‘agree’, score 4/5)

### Theme 10: Uncertainty about prescribed medication

Although expressing worry about potential side-effects, some participants chose the ‘uncertain’ response option when answering concern items to reflect their own uncertainty about their medication and how these medications worked. This underestimated their medication concern beliefs.

“…I would probably say uncertain at the moment…what makes me feel uncertain about it is, you know, what other side-effects it could pose…could it make my psoriasis worse if I ever stop taking it…*I do worry* whether if I ever stopped the medication, could something trigger it off to come back much, much worse than it was before. So that’s a worry.” [P3, specific concerns Q6 ‘Having to take my psoriasis medications worries me’, item response ‘uncertain, score 3/5].

### General overuse and harmfulness domains

The analysis produced one theme for the general concern domains, relating to item misinterpretation.

### Theme 11: Medication-specific

Only one item in the general overuse and harmfulness scales did not generate any problems (‘If doctors had more time with patients they would prescribe fewer medicines’). Participants found items in these scales difficult to answer, as they thought their answer depended on further information about the medication that was not specified in the question, such as type of medication and how it was to be used.

“I’m going to say uncertain about that and again because it really depends on what type of *medicine* you are talking about and what you are *using* it for.” [P4, general concerns Q17 ‘Most medicines do more harm than good’, item response ‘uncertain’, score 3/5]

## Discussion

This qualitative study examined the extent to which the BMQ is suitable for assessing condition-specific medication beliefs, using psoriasis as a suitable exemplar of a complex condition characterised by relapsing-remitting symptoms and considerable medication challenges. Psoriasis was a useful condition to choose as its treatment includes a range of therapeutic approaches including different mechanisms. Its treatment pathway shares a great deal with other inflammatory conditions such as rheumatoid arthritis, inflammatory bowel disease as well as other long-term dermatological conditions. This work has important implications for the use of the BMQ in research and clinical settings.

### Personal model of psoriasis and medication

Individuals’ perceptions of their psoriasis and medication influenced the extent to which the BMQ items were able to capture the aspects of the constructs they were designed to measure. Consistent with previous research [28, 29], psoriasis was viewed as a condition that was separate from perceptions of illness and disease, with a wide impact on functioning and complex medication outcomes. Due to the mismatch between the terminology used in the scale and individuals perceptions of psoriasis, the existing BMQ items led to scores that underestimated levels of medication necessity beliefs. Furthermore, the existing BMQ items were too broad to capture the complexity of participants’ perceptions of psoriasis management and medication outcomes, resulting in scores being an inaccurate representation of medication necessity beliefs.

### Misinterpretation

Many of the misinterpretation problems identified in this study are parallel to those reported in other LTCs with different psychometric tools [33–36], with participants experiencing difficulty in generating an answer or answering different questions from what was asked. However a crucial key discovery was that the levels of medication concerns held by participants were significantly underestimated when assessed using the BMQ. Although some participants had stopped taking their prescribed psoriasis therapy because of their concerns about its usage, they believed the concern statements no longer applied *because* they were no longer using the medication.

Some BMQ items did not generate any problems with item content and/or misinterpretation, whilst others produced multiple problems. It was not the aim at this stage to provide a quantitative assessment of the number of problems generated for each individual item, but to identify general and condition-specific difficulties experienced by individuals when completing the BMQ. Indeed, it is clear that many of the problems were applicable to *all* items within each BMQ domain.

### Medication adherence and supporting shared decision-making

The introduction of the Necessity-Concerns Framework by Horne and colleagues has changed our understanding of the role of medication beliefs in medication adherence. However there are differences in effect sizes in the association between medication beliefs and adherence, with some studies reporting no association [13, 14]. Whilst the BMQ has been a major contribution to the assessment of medication beliefs, this study has identified limitations of its use in complex LTCs. There is increasing recognition that we need to increase the specificity of measurement tools for the condition, for example, the authors of the tool most commonly used to assess illness beliefs, the Illness Perception Questionnaire-Revised [40], encourage the adaptation of the scale to different condition- or disease contexts. This approach can only help improve the validity of the core tools needed to assess predictors of adherence behaviours. Future work should examine the suitability of the BMQ in other chronic illness populations, as many of the problems identified in this study are applicable to other illness groups.

Many patients perceive healthcare professionals, especially GP’s, as lacking expertise, empathy and understanding in managing psoriasis, with some patients with poorly controlled psoriasis finding it difficult to obtain a specialist dermatology referral from their GP and some withdrawing from healthcare professional support [28, 29]. In addition, there is evidence to suggest that a large proportion of people with psoriasis believe that their condition is not treated sufficiently aggressively [26]. This study has taken this further by demonstrating that participants who were being managed by their GP expressed strong beliefs that access to psoriasis medications is overly restrictive. An important area for future investigation is to assess the relationship between these illness-specific beliefs with medication adherence; for individuals who are being managed in primary care with topical therapies, individuals may *underuse* their topical medication in order to be referred to specialist care for systemic therapies. Alternatively, individuals may *overuse* their topical medication in an attempt to manage uncertain topical medication efficacy and gain control over the problematic control of symptoms. A belief that medications are overused is a common concern [41] and captured in the BMQ (General-overuse subscale). The addition of a subscale representing beliefs in the overly restrictive access to medication is required if researchers and clinicians are to be able to identify the full range of beliefs that influence medicines adherence.

In the UK NICE Medicines Adherence Guidelines [7], there is strong emphasis on the need to explore patients’ perspectives of medicines, to encourage patients to discuss any doubts or concerns they have about their medication and whether they believe they need them, in order to help ensure that people actively participate in decision-making and to support optimal self-management. Patients do not openly disclose their beliefs to clinicians [42], through fear of being labelled a ‘bad patient’, undeserving, and through fear of potential negative consequences (e.g. being taken off their treatment). Self-report tools may provide a valuable aid for clinicians in helping to elicit patients’ perceptions of their medication. We have demonstrated the challenges of assessing medication beliefs in a common and complex LTC, including the need to ensure that tool items are able to represent condition-specific beliefs and are clear to the individual in order to prevent item misinterpretation.

### Strengths and limitations

We acknowledge that the total number of participants was small, with the majority recruited through support groups, and thus the findings may only be generalisable to this group. However recruitment from non-clinical settings resulted in a diverse sample ranging in disease and treatment history, including those who have disengaged from conventional health services. The nature of recruitment precludes assessment of the reasons given for non-participation. Although participants were not involved in the design of this study, all participants were fully briefed on its aims, interpretation and implications. Due to the nature of the study, participants did not check the transcripts. This is because participants may have requested to change their ‘real time’ response if, for example, they initially misinterpreted the items. Asking participants using combination therapy to complete the BMQ twice may have increased the cognitive complexity of the task. Although we acknowledge this limitation, it realistically reflects the inherent challenges of measuring medication beliefs in individuals managing complex LTCs and polypharmacy. Our results are broadly consistent with other think-aloud studies using different psychometric tools and clinical populations [33–36], with the sample size similar to other think-aloud studies [34, 36]. Although the think-aloud method has provided a valuable technique for revealing the challenges of assessing complex medication beliefs, future studies should develop this tool using additional psychometric evaluations.

This is the first study to explore the complexity of assessing medication beliefs in ‘real time’. The Necessity-Concerns Framework continues to have a pivotal role in furthering our understanding of medication adherence, however this study has identified problems with the items intended to measure is central constructs. We recommend that the BMQ is adapted by researchers for disease or medication specific use, perhaps by using real-time assessments of participants’ responses. The current study has emphasised the importance of adapting the BMQ to represent condition-specific medication beliefs, including focusing on the terminology used to describe the condition under investigation, considering the complexity of illness outcomes, and the need to identify *additional* condition-specific medication beliefs, such as beliefs in overly restrictive access to medications. It is also necessary to ensure that the statements are unambiguous to participants in order to prevent item misinterpretation, which may include terminology changes, providing clarifying statements, or changing item response choices. Suggested condition-specific and general changes are presented in Table 6.

**Table 6:** Condition-specific and general changes for the BMQ

Item content	Condition-specific changes	General changes to instructions/terminology
Specific necessity	Ensure that terminology used to describe the condition maps onto participants’ perceptions of their condition.	A clarifying statement should precede specific necessity items. E.g. ‘*Please refer to your personal views about the impact of the medicines on your overall future health*, not the impact of potential medication side-effects on future health’
	Need to differentiate between the impact of medication on physical symptoms and psychological/social well-being	A clarifying statement should precede specific necessity items. E.g. ‘You may be using a different medication in the future. However *please refer to your personal views about the medication currently prescribed for you’*
Specific concerns	Need to differentiate between: 1.Short- *and* long-term medication side-effects 2.Current *and* future medication disruptions to daily life 3.Physical *and* psychological medication dependency	A clarifying statement should precede specific concern items. E.g. ‘Some people choose not to use their medication as prescribed. However *please imagine that you are using your medication as prescribed and indicate whether this would make you experience feelings of worry’*
	Identify additional condition-specific beliefs (e.g. overly restrictive access to medications)	A clarifying statement should precede specific concern items. E.g. *‘Please refer to your personal views about your medicines,* not your condition’
		The specific concern item ‘My psoriasis medications are a mystery to me’ should be rephrased. E.g. ‘I sometimes experience uncertainty about my medicines and how they work’
General concerns		Ensure the phrase ‘in general’ precedes each item. E.g. *‘In general, doctors use too many medicines’*
Item response choice	Condition-specific changes	General changes to instructions/terminology
All BMQ domains		For all BMQ items, the item response choice ‘uncertain’ should be changed to ‘don’t know’

## Conclusions

Using psoriasis as a suitable exemplar of a complex condition associated with considerable medication challenges, this study has identified general and condition-specific difficulties experienced by individuals completing the BMQ in ‘real time’. Medication beliefs were underestimated, or not fully captured, by existing BMQ items. In order to improve our understanding of medication adherence and help support shared decision-making, the results from this study should be used as a basis in the development of condition-specific BMQ items, for use in both a research and clinical setting.

## Abbreviations

BMQ: Beliefs about medicines questionnaire
GP: General practitioner
LTC: Long-term condition
NICE: National Institute for Health and Care Excellence
WHO: World Health Organisation
